# Determination of Relationships between Symmetry-Based, Performance-Based, and Functional Outcome Measures in Patients Undergoing Total Hip Arthroplasty

**DOI:** 10.3390/jpm13071046

**Published:** 2023-06-26

**Authors:** Jana Kirschner, Sven Michel, Roland Becker, Olaf Stiebitz, Hagen Hommel, Robert Schulz, Maciej Daszkiewicz, Aleksandra Królikowska, Robert Prill

**Affiliations:** 1 Department of Therapy Sciences, Brandenburg University of Technology Cottbus-Senftenberg, 03046 Cottbus, Germany; sven.michel@b-tu.de (S.M.); olafstiebitz@lebenslang-lernen.eu (O.S.); h.hommel@khmol.de (H.H.); 2 Center of Orthopaedics and Traumatology, University Hospital Brandenburg a.d.H., Brandenburg Medical School Theodor Fontane, 14770 Brandenburg an der Havel, Germany; roland_becker@yahoo.de; 3 Faculty of Health Sciences Brandenburg, Brandenburg Medical School Theodor Fontane, 14770 Brandenburg an der Havel, Germany; 4 District Hospital Märkisch Oderland GmbH, 16269 Wriezen, Germany; 5 BIH QUEST Center for Responsible Research, Berlin Institute of Health at Charité, Universitätsmedizin Berlin, 10178 Berlin, Germany; robert.schulz@bih-charite.de; 6 eMKaMED Medical Center, 53110 Wroclaw, Poland; daszkiewiczmaciej98@gmail.com; 7 Ergonomics and Biomedical Monitoring Laboratory, Department of Physiotherapy, Faculty of Health Sciences, Wroclaw Medical University, 50367 Wroclaw, Poland

**Keywords:** biomedical monitoring, hip replacement, measurement set, outcome set, reliability, performance-based measures

## Abstract

Due to the high heterogeneity in outcome measures after total hip arthroplasty (THA), the prospective observational study investigated the relationships between symmetry-based (SBMs), performance-based (PBMs), and functional outcome measures in THA patients to determine necessary or redundant categories of tests. The study material consisted of 24 patients with end-stage hip osteoarthritis scheduled for THA. The patients were examined one day before surgery and consecutively on the 4th day, 9th day, and 10th week postoperatively using the SBMs (weight-bearing chair-rising test, measurements of the maximal isometric torque of the quadriceps muscle); the PBMs (10-m walk, timed up-and-go, and stair-climbing tests); and the functional outcome measure (Harris Hip Score). The results obtained in a given category of tests at different time points were compared, and the correlations between the tests were determined. The reliability of the outcome measures was determined. The results of tests in the studied categories statistically significantly (*p* < 0.05) improved at the 10th week postoperatively compared to preoperative results. No strong correlations were revealed between the three studied types of outcome measures in THA patients. Therefore, none of them can be considered redundant. It also means that the relevance of symmetry for a core measurement set to describe the domain function in THA patients must be further clarified.

## 1. Introduction

There is a need to improve reporting standards in orthopedics [1]. Currently, there are very heterogeneous statements on outcome measures for patients with total knee (TKA) or total hip (THA) arthroplasties [2,3]. Diverse outcomes, like balance aspects or muscle strength measured under isokinetic conditions, are used to describe the recovery and performance of patients after the THA [4,5]. Also, different outcome assessment scores are largely used to assess functional capacity in patients after THA or TKA [6,7,8]. Whether the function of the hip joint can be comprehensively evaluated with the help of questionnaires remains debatable. One of the most commonly used questionnaires for THA patients is the Harris Hip Score (HHS) [9].

Often, there is inconstancy concerning the follow-up time points, the measurement tools, and the whole idea of how ‘function’ should be defined. Reliable and valid test instruments specifically for postoperative THA treatment are needed to assess therapy success objectively. In recent years, there have been intensive efforts to close these gaps. For this purpose, the Initiative for Method, Measurement, and Pain Assessment in Clinical Trials (IMMPACT) and the Outcome Measures in Rheumatology (OMERACT) try to determine core outcome set (COS) and core measurement sets to help choose assessments for the domain physical function after THA. Their tasks include refining physical function measurements, including an evaluation with PROMs and performance-related measures [10]. According to Singh et al. (2015), there is, for now, no single measurement tool to evaluate surgical outcomes after THA or TKA. The heterogeneous different examination tools make it challenging to compare postoperative results [11].

Performance-based measures (PBMs), such as sit-to-stand transfer, timed up-and-go test, or stair-climbing test, are practical assessments for the objective representation of the domain functions of the lower extremity [12,13,14]. The role of symmetry and the redundance of established functional assessment tools and PBMs against each other must be discussed. Therefore, the present study investigates the relationships between symmetry-based, performance-based, and functional outcome measures in THA patients to determine necessary or redundant categories of tests. It was hypothesized that the symmetry-based, performance-based, and functional outcome measures do not correspond with each other among this group of patients.

## 2. Materials and Methods

The prospective, observational cohort study was conducted in a highly specialized hospital for arthroplasty. It was reviewed and approved by the local medical chamber under the number S3(a)/2017. Informed consent was obtained from all subjects involved in the study. The study had a repeated-measures design.

The studied material consisted of patients with end-stage osteoarthritis (OA) of the hip joint who were scheduled for THA. There were the first 24 patients who met all inclusion criteria: being scheduled for primary THA; end-stage hip OA; age > 60 years; ability to walk more than 25 m before THA; not living in a nursing home. Exclusion criteria were present and/or previous musculoskeletal disorders other than hip OA; current injuries of the lower extremities or trunk; cardio-pulmonary diseases with reported influence on limiting performance; or any diseases affecting balance like vestibular or brain-affecting diseases.

All patients were operated on by a senior main surgeon from a certified arthroplasty center. The operation was performed via a minimally invasive anterolateral approach. All endoprostheses were implanted uncemented. There were no intraoperative or postoperative complications during the observation period. The postoperative radiological control of the position of the prosthesis did not reveal any abnormalities.

The following categories of tests were used to assess the physical function of the hip joint: (1) symmetry-based measures, including weight-bearing chair-rising tests (CRT) and measurements of the maximal isometric torque (IT) of the quadriceps muscle; (2) performance-based measures: 10-m walk test (10 MWT), timed up-and-go test (TUG), stair-climbing test (SCT); (3) functional outcome measures: HHS.

### 2.1. Symmetry-Based Measures

#### 2.1.1. Weight-Bearing Chair-Rising Test

The weight-bearing CRT was carried out with simultaneous measurement of ground reaction forces using two force plates separately for involved and uninvolved limbs (Leonardo Mechanograph® GRFP STD and Leonardo Mechanography v. 4.4 Software, Novotec Medical GmbH, Pforzheim, Germany). The examined patient was instructed to stand up from the bench, take a complete stance, and sit down again. There were two repetitions of the test. The measured parameter was the between-limbs difference, expressed in kilonewtons (kN).

#### 2.1.2. Measurement of the Maximal Isometric Strength of the Quadriceps Muscle

The measurement of the maximal isometric strength of the quadriceps muscle was performed using a hand-held dynamometer (MicroFet 2, Hogan Health Industries, Inc., West Jordan, UT, USA). The torque was measured bilaterally, starting from the uninvolved limb. The measurements were carried out in a seated position with the examined limb flexed to 90 degrees. The hand-held dynamometer was held by the examiner proximally to the ankle joint. The examined patient was instructed to perform maximal-effort contraction of the quadriceps muscle against resistance applied by the examiner. The resistance had to be large enough to make extending the knee on the examined side impossible. The measured parameter was a peak expressed in Newtons (N).

### 2.2. Performance-Based Measures

#### 2.2.1. Ten-Meter Walk Test (10 MWT)

The 10 MWT assessed the time a patient walked a distance of 10 m. To ensure that possible starting and stopping distances did not influence the measurement results, the patient started and ended 2 m before and after the actual test distance of 10 m. If necessary, the patient could use a walking aid during the 10 MWT, but this had to be noted in the documentation. Verbal commands were used. The measurement was carried out using a photoelectric sensor and stopwatch. The measured parameter was time, expressed in seconds (s).

#### 2.2.2. Timed Up-and-Go Test (TUG)

The TUG test assessed the time required for a patient to rise from a standardized chair with a seat height of 46 cm, walk three meters at a comfortable pace to a mark placed on the floor, turn around at the three meters mark, walk back to the starting point, and finally, return to sitting in the chair. During the TUG test, the patient could use a walking aid, if necessary, although it had to be recorded in the documentation. Verbal commands were used. The measurement was carried out using a stopwatch. The measured parameter was time, expressed in seconds (s).

#### 2.2.3. Stair-Climbing Test (SCT)

The SCT evaluates whether and in how much time the patient can walk up and down a given number of stairs. As the 9-step SCT was found to be very sensitive to initial deterioration and improvement in the early phase of THA, a 14-step stair-climbing test was selected [15]. Preoperatively, the test was carried out with no walking aids. Postoperatively, walking support and the stair railing were used. When going up the stairs, the patient placed the uninvolved limb first. When going down the stairs, the patient placed the involved limb first. The measurement was carried out using a stopwatch. The measured parameter was time, expressed in seconds (s).

### 2.3. Functional Outcome Measures

#### Harris Hip Score

The HHS was collected in the German version of Fortbildungen für Orthopädische Medizin und Manuelle Therapie (FOMT). The HHS assesses patients in terms of pain (one item, 0–44 points), function (seven items, 0–47 points), absence of deformity (one item, 4 points), and range of motion (two items, 5 points), giving a total score out of 100 [9]. The so-called function domain consists of daily activities, including stair use, using public transportation, sitting and managing shoes and socks, and gait, including limp, support needed, and walking distance.

Patients were assessed at four defined time points, as presented in Table 1, precisely T0, one day before surgery; T1, on the 4th postoperative day (3.96 ± 0.95 days); T2 on the 9th postoperative day (8.62 ± 0.77 days); T3, at the 10th (9.29 ± 1.71 weeks) week postoperatively.

### 2.4. Reliability of the Outcome Measures

The reliability of all symmetry-based and performance-based measures was established at every time point, namely T0, T1, T2, and T3. All patients in the studied group performed the given tests twice on the same day. According to the methodology described in Section 2.1 and Section 2.2 of the present paper, The tests were administered by the same examiner. The reliability and validity of HHS in THA patients have already been proven; therefore, it was not included in the present study [16,17].

### 2.5. Statistical Analysis

Due to missing pilot values, the sample-size calculation was carried out power-based, and following a similar study on TKA [18], with the help of G-Power 3.1.9.2, assuming mean effects of 0.5, p 0.05, and a power of 80%, 24 patients were required for sufficient correlation analysis.

The anonymized raw data, which were received in the form of data protocols and completed questionnaires, were transferred to an Excel spreadsheet and then imported into “R” and evaluated with the statistics programs “R (Version 3.4.4)” and “R-Studio (Version 1.1.442)”. The study assumed a comparison of the results obtained in a given category of tests at different time points and correlations between the tests at different time points.

The arithmetic mean (x) and standard deviation (±) of the studied parameters were calculated for the studied group of patients. The normal distribution of the studied parameters was determined using the Shapiro–Wilk test.

The assessment results with the use of HHS, a total score of fewer than 70 points, was considered a poor result; 70–80 points a fair result; 80–90 points a good result, and 90–100 points an excellent result [19].

According to the results of the Shapiro–Wilk test, the results of the performed measures involved in three different categories obtained at T0 were compared to results reported at T3 using parametric or nonparametric t-tests for dependent samples. The level of significance was defined as *p* < 0.05.

The linear Pearson’s correlation coefficient (*r*) for normally distributed data and Spearman’s correlation coefficient for not normally distributed data were calculated to measure any relationships between the results of the tests belonging to three different categories at all time points. The magnitudes of all of the bivariate associations were classified as negligible (0.00–0.30), low (0.31–0.50), moderate (0.51–0.70), high (0.71–0.90), and very high (0.91–1.00) [20]. Additionally, the coefficient of determination, the *r*-squared (*r*^2^), was calculated to give a proportion of the variance of one variable that is predictable from the other variable. In other words, *r*^2^ represents the percentage of data points closest to the line of best fit.

The relative reliability assessment of each test was based on the intraclass correlation coefficient (ICC) calculation according to the guidelines described by Shrout and Fleiss (1979) [21]. As the intra-rater test reliability was determined, a two-way mixed-effects model, single measurement type, and absolute agreement definition were used [22]. Values less than 0.50 indicated poor reliability; values between 0.50 and 0.75 demonstrated moderate reliability; values between 0.75 and 0.90 were determined to have good reliability; and values greater than 0.90 indicated excellent reliability [22].

## 3. Results

The final sample of 24 patients included 18 males and six females. The mean age of the patients was 63.5 ± 8.3 years (males 64.6 ± 7.6 years; females 60.0 ± 10.0 years), and a mean body mass exceeded 89.2 ± 15.97 kg. The mean Body Mass Index amounted to 30.0 ± 4.3 kg/m^2^ (men 30.1 ± 4.6 kg/m^2^; women 30.0 ± 3.90 kg/m^2^).

### 3.1. Symmetry-Based Measures

**Figure 1:**
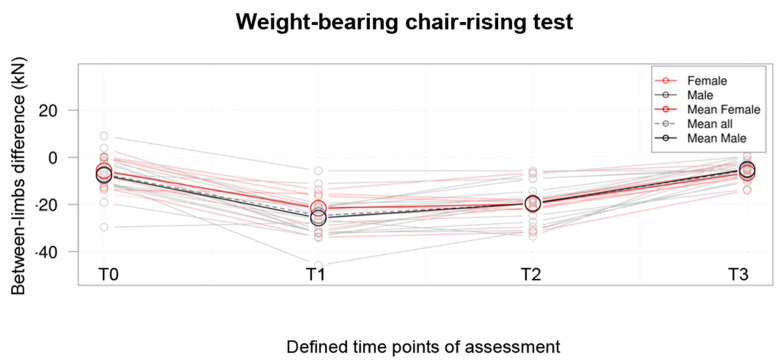
Comparison of the weight-bearing chair raising test with simultaneous measurement of ground reaction forces results obtained at the four defined time points, namely T0, one day before surgery; T1, on the 4th postoperative day; T2, on the 9th postoperative day; T3, at the 10th week postoperatively.

The postoperative result of the weight-bearing CRT with simultaneous measurement of ground reaction forces statistically significantly improved (*p* < 0.05) compared to the preoperative result, as presented in Figure 1. The between-limbs difference at T3 that exceeded x = −5.47 ± 4.01 kN was reduced compared to the between-limbs difference noted at T0, amounting to x = −7.13 ± 8.42 kN.

**Figure 2:**
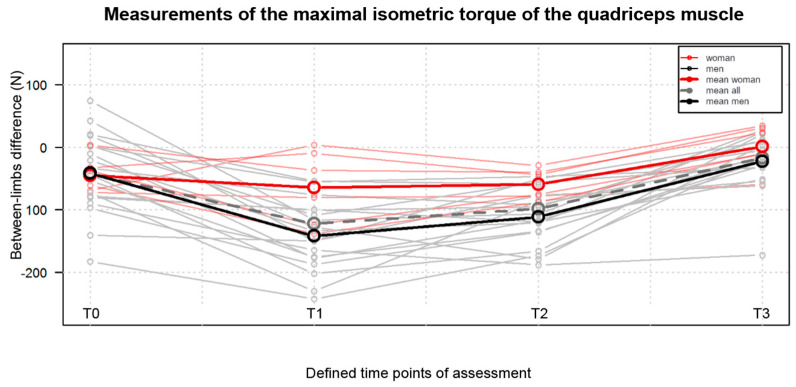
Comparison of the results of the measurement of the maximal isometric strength of the quadriceps muscle obtained at the four defined time points, namely T0, one day before surgery; T1, on the 4th postoperative day; T2, on the 9th postoperative day; T3, at the 10th week postoperatively.

Also, the between-limb symmetry regarding maximal IT of the quadriceps muscle improved postoperatively compared to preoperative results, as presented in Figure 2. The between-limbs difference at T3, which amounted to x = −16.71 ± 44.34 N, was reduced when compared to the between-limbs difference noted at T0, amounting to x = −42.18 ± 11.52 kN.

### 3.2. Performance-Based Measures

**Figure 3:**
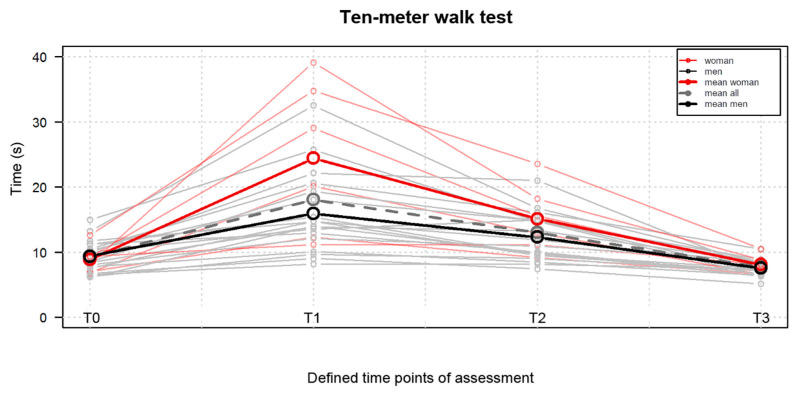
Comparison of the ten-meter walk test results obtained at the four defined time points, namely T0, one day before surgery; T1, on the 4th postoperative day; T2, on the 9th postoperative day; T3, at the 10th week postoperatively.

The results of the 10 MWT statistically significantly (*p* < 0.05) improved at T3 (x = 7.67 ± 1.30 s) when compared to T0 (x = 9.24 ± 2.38 s), as shown in Figure 3.

**Figure 4:**
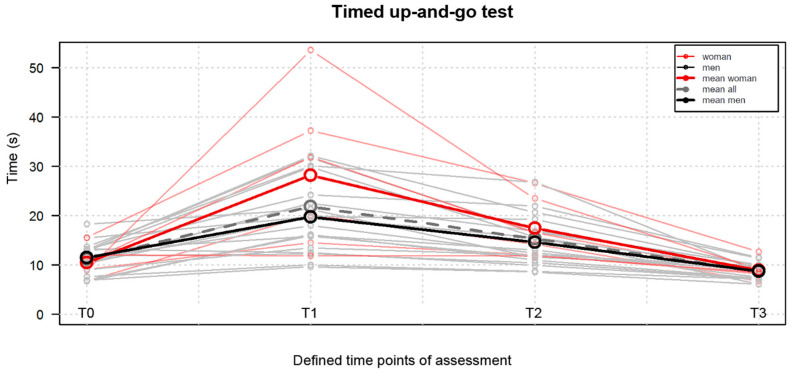
Comparison of the timed up-and-go test results obtained at the four defined time points, namely T0, one day before surgery; T1, on the 4th postoperative day; T2, on the 9th postoperative day; T3, at the 10th week postoperatively.

A statistically significant improvement (*p* < 0.05) was also reported for the TUG results. The time to perform the test decreased at T3, amounting to x = 8.74 ± 1.72 s, from T0, amounting to x = 11.22 ± 3.11 s. The results are presented in Figure 4.

**Figure 5:**
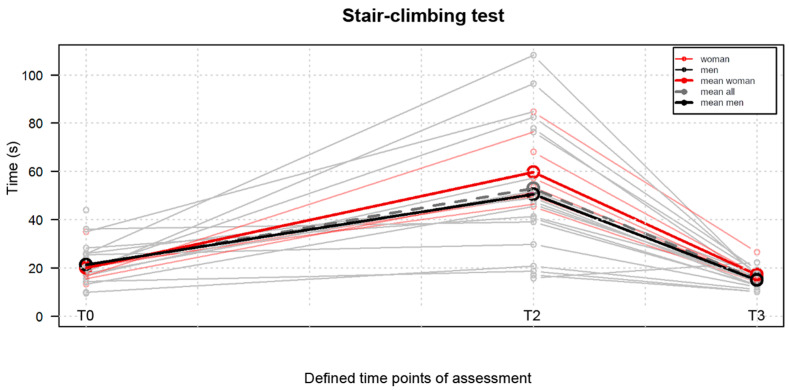
Comparison of the stair-climbing test results obtained at the three defined time points, namely T0, one day before surgery; T2, on the 9th postoperative day; T3, at the 10th week postoperatively.

Comparable to the two above performance-based measures, the SCT results were also statistically significant (*p* < 0.05) better postoperatively than preoperatively, as presented in Figure 5. The time to perform the test noted at T3 exceeded x = 15.51 ± 3.72 s was decreased when compared to T0 when it amounted to x = 21.00 ± 8.36 s.

### 3.3. Functional Outcome Measures

The total HHS statistically significantly (*p* < 0.05) improved from a poor result (x = 48.25 ± 12.04 points) at T0 to a good result (x = 87.54 ± 11.91 points), reported at T3. The pain domain statistically significantly (*p* < 0.05) improved from x = 12.92 ± 4.64 points at T0 to x = 39.83 ± 7.87 points at T3. A statistically significant (*p* < 0.05) improvement was also reported for the daily activities domain, which increased from x = 7.79 ± 3.19 points at T0 to x = 12.25 ± 1.82 points at T3. Also, the domain of gait statistically significantly (*p* < 0.05) improved from x = 21.12 ± 5.97 points at T0 to x = 27.12 ± 4.40 points at T3. A minor improvement was reported regarding the absence of deformity as the results obtained at T0 and T3 amounted to x = 3.21 ± 1.10 points and x = 3.92 ± 0.41 points, respectively. The domain of range of motion statistically significantly (*p* < 0.05) improved from x = 3.21 ± 0.83 points at T0 to x = 4.33 ± 0.64 points at T3.

### 3.4. Correlations between the Categories of Tests

**Figure 6:**
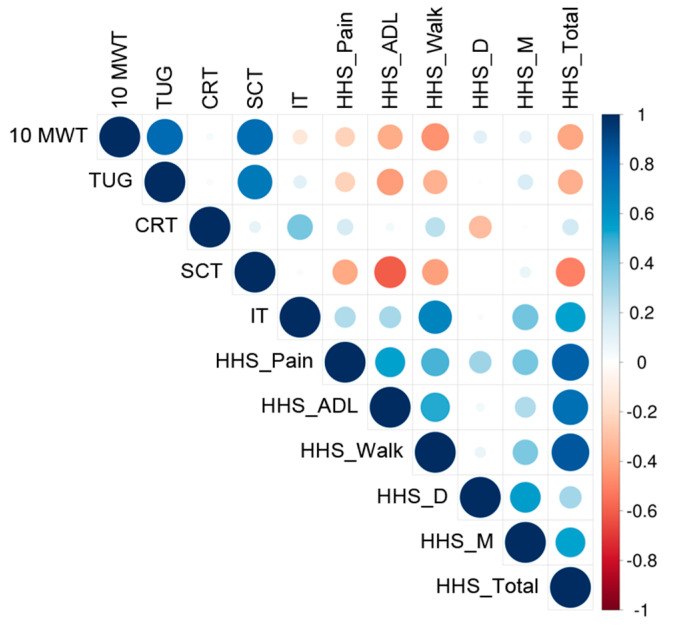
Correlations between the results of the tests performed at the 10th week postoperatively representing three different categories of measurements, namely symmetry-based measures: CRT, weight-bearing chair raising test with simultaneous measurement of ground reaction forces; IT, measurements of the maximal isometric torque of the quadriceps muscle; performance-based measures: 10 MWT, 10-m walk test; TUG, timed up-and-go test; SCT, stair-climbing test; functional outcome measures: HHS, Harris Hip Score, including pain (HHS_Pain), activities of daily living (HHS_ADL), gait (HHS_Walk), absence of deformity (HHS_D), and range of motion (HHS_M).

Despite an enormous number of possibilities between all the studied variables at all time points, no high nor very high correlations were noted (*r* < 0.70). In Figure 6, there were correlations at the last time point.

A moderate negative correlation was noted between the HHS gait domain at T0 and 10 MTW at T3 and (*r* = −0.60). The better score in the HHS gait domain preoperatively, the shorter time to cover the 10 MWT at the 10th week postoperatively. The 36% of the total variation in time to cover the 10 MWT at the 10th week postoperatively can be explained by the negative relationship between the 10 MWT at T3 and obtained HHS gait domain score at T0 (*r*^2^ = 0.36). The other 64% of the total variation remains unexplained.

Also, a moderately negative correlation was recorded for the SCT at T0 and HHS activities of a daily living domain at T3 (*r* = −0.65). The SCT at T0 was also moderately negatively correlated with the HHS gait domain at T3 (*r* = −0.61). The shorter the time to cover the SCT preoperatively, the better the score in HHS activities of daily living and the HHS gait domain at the 10th week postoperatively. The 42% of the total variation in HHS activities of a daily living domain at T3 can be explained by the negative relationship between the SCT at T0 and HHS activities of a daily living domain at T3 (*r*^2^ = 0.42). Similarly, 37% of the total variation in the HHS gait domain at T3 can be explained by the negative relationship between the SCT at T0 and the HHS gait domain at T3 (*r*^2^ = 0.37). The other 58% and 63% consecutively remain unexplained.

Next, the SCT at T3 was moderately negatively associated with the HHS gait domain at T3 (*r* = −0.60). In the 10th week postoperatively, the shorter time to cover SCT, the better score in the HHS gait domain. The 36% of the total variation in time to cover the SCT can be explained by the negative relationship between the SCT and HHS gait domain (*r*^2^ = 0.36). The other 64% of the total variation remains unexplained.

A moderately negative correlation was noted between the difference in maximal IT of the quadriceps muscle in the involved and uninvolved limbs at T0 and the HHS gait domain at T3. The *r*^2^ ranged from 0.36 to 0.42. From 36% to 42% of the total variation in the HHS gait domain at the 10th week postoperatively can be explained by the negative relationship between the difference in maximal IT of the quadriceps muscle in the involved and uninvolved limbs at T0 and the HHS gait domain at T3. The other 64% and 58% consecutively remain unexplained.

The rest of the correlations were lower than moderate.

### 3.5. Reliability of the Outcome Measures

As presented in Table 2, the symmetry-based measures were characterized by excellent reliability, with the ICC ranging from 0.962 to 0.989.

The values of the ICC for the performance-based measures ranged from 0.902 to 0.995, indicating excellent reliability, as presented in Table 3.

## 4. Discussion

The present study investigated the relationships between symmetry-based, performance-based, and functional outcome measures in THA patients. There was no strong relationship between the three studied categories of tests. Therefore, it was proven that the symmetry-based, performance-based, and functional outcome measures do not correspond with each other among this group of patients. Because of that, no categories of tests were considered redundant.

With a prevalence of 10% in people over 55 years of age, OA is the most common joint disease, causing significant pain and disability [23]. THA is undertaken to relieve pain and improve function in patients with advanced hip OA whose symptoms are impossible to manage with conservative treatment. The procedure is one of the most successful in orthopedics, although it is also one of the most cost-effective. The number of THAs performed is increasing every year [24]. The evaluation of THA patients after the intervention is critical. The hip joint is one of the most stable but, at the same time, mobile joints of the lower limbs. The function of the hip joint is based primarily on the muscular contribution to support during weight-bearing movements that result in significant loads and stress on cartilage and soft tissues [25].

Of course, it is worth keeping in mind that the outcome measures might be considered an artificial construct as the life and well-being of the patients are influenced by many other factors than a specific illness or treatment. Patients after THA should be regularly put through different measures to monitor their clinical and functional outcomes, evaluate their progress, and introduce eventual changes in postoperative physiotherapeutic procedures. The follow-ups also define the THA’s final clinical and functional effects. Moreover, constant outcome measures remain reliable for detecting early signs of failure. It is vital in light of early intervention, as the extensive bone damage makes revision surgery more difficult and significantly increases treatment costs [26].

The results of the individual tests taken together always constitute a large amount of data. Therefore, for another group of examined patients, the authors proposed a strategy that decreased the number of data points to be analyzed without reducing the number of tests performed, like composite scores [27,28]. However, considering the demographics of THA patients, it seems more rational to identify the measures of the highest importance.

The study did not reveal any strong relationships between the studied outcome measures. Some correlations were noted on a moderate level; however, they should be analyzed cautiously. It must be highlighted that only 36% to 42% of the total variation can be explained by those correlations (*r*^2^ = 0.36–0.42). That means the other 58–64% of the total variation remains unexplained.

The study has also determined the reliability of the utilized symmetry-based and performance-based measures. Reliability refers to the extent to which results of a given measure for patients whose status has not changed are reproducible. The reliability can be assessed under several conditions. Presented in the given study, test-retest represents testing over time. Retesting can be performed by different raters on the same occasion (inter-rater reliability) or by the same rater on different occasions (intra-rater reliability) [29]. It’s crucial in orthopedics and physiotherapy that the outcome measures must be evaluated regarding their reliability, validity, and responsiveness [30,31,32,33]. For the present study purposes, the reliability of the symmetry-based and performance-based measures was determined separately for each defined point of assessment. It was determined that symmetry-based measures (weight-bearing chair raising test with simultaneous measurement of ground reaction forces, measurement of the maximal isometric strength of the quadriceps muscle), as well as performance-based measures (ten-meter walk test, timed up-and-go test, stair-climbing test), are characterized by excellent reliability. The reliability of the HHS was not assessed as it had already been done by other authors [16,17].

One of the limitations of the study was the unequal number of male and female patients in the study group. It has been highlighted in the literature that the relationship between sex and gender-related factors and postoperative outcome and satisfaction in patients undergoing THA is unclear, and more studies on this issue are needed [34]. Therefore, it is hard to judge how the inequality of men and women in the studied group could affect the results.

It also needs to be emphasized that in the final decision on which performance- or time-based measurement to include, a larger sample with subgroups in terms of gender, activity level, and severity of symptoms prior to surgery might be necessary. The authors support the idea of stratification in terms of those confounders for interventional studies. For the observational character of this study in terms of outcome progress, neither the exclusion of all potential confounders nor the exact power analysis seems crucial. Still, the authors agree that the low power of the study, due to a small sample size, constitutes a significant limitation.

Another limitation might be the usage of only HHS regarding functional outcomes, as it is a clinician-based questionnaire designed to be administered by a healthcare professional. In the future, it would be good to include patient-reported outcome measures, as clinician-based tools may not accurately reflect the perception of the patient following the intervention [35]. Therefore, patient-reported outcome measures constitute a gold standard in evaluating patients with musculoskeletal disorders, whose perspective and health-related quality of life are important [9]. Next to the HHS that was utilized in the present study, the most commonly used functional outcome measures primarily developed for patients after THA include the Merle d’Aubigné and Postel Hip Score [36]. However, the score is also a clinician-based one. The patient-based questionnaires also have an added value: they don’t need to be administered by a trained healthcare specialist [37]. Other functional outcome measures dedicated to the hip joint are the Hip Disability and Osteoarthritis Outcome Score (HOOS) and the Oxford Hip Score (OHS) [9]. Additionally, sometimes the general status of health is assessed, commonly with the use of Short Form 36 (SF-36) [38]. However, the undoubted value of using HHS is that it is widely used in the orthopedic literature; therefore, comparing the obtained results with other studies is more manageable.

## 5. Conclusions

The study revealed no strong relationships between body symmetry-based, performance-based, and patient-reported outcome measures in THA patients. Therefore, none of the studied categories of tests can be considered redundant. It also means that the relevance of symmetry for a core measurement set to describe the domain ‘function’ in THA patients must be further clarified.

## Figures and Tables

**Table 1:** The scheme of measures that were performed at four defined time points.

Category	Measure	T0	T1	T2	T3
Symmetry-based measures	Weight-bearing chair-rising test (CRT)	x	x	x	x
	Isometric torque (IT)	x	x	x	x
Performance-based measures	Ten-meter walk test (10 MWT)	x	x	x	x
	Timed up-and-go test (TUG)	x	x	x	x
	Stair-climbing test (SCT)	x	-	x	x
Functional outcome measures	Harris Hip Score (HHS)	x	-	-	x

T0, one day before surgery; T1, on the 4th postoperative day (3.96 ± 0.95 days); T2 on the 9th postoperative day (8.62 ± 0.77 days); T3, at the 10th (9.29 ± 1.71 weeks) week postoperatively.

**Table 2:** The test-retest reliability results of the symmetry-based measures at the four defined time points.

Symmetry-Based Measures					
Measure	Studied Limb	T0	T1	T2	T3
Weight-bearing chair-rising test (CRT)	Involved	0.964	0.962	0.938	0.970
	Uninvolved	0.980	0.976	0.970	0.970
Isometric torque (IT)	Involved	0.981	0.989	0.986	0.986
	Uninvolved	0.986	0.973	0.978	0.988

The values are expressed as the intraclass correlation coefficient. T0, one day before surgery; T1, on the 4th postoperative day; T2, on the 9th postoperative day; T3, at the 10th week postoperatively.

**Table 3:** The test-retest reliability results of the performance-based measures at the four defined time points.

Performance-Based Measures				
Measure	T0	T1	T2	T3
Ten-meter walk test (10 MWT)	0.962	0.951	0.967	0.929
Timed up-and-go test (TUG)	0.964	0.981	0.973	0.961
Stair-climbing test (SCT)	0.995	-	0.902	0.966

The values are expressed as the intraclass correlation coefficient. T0, one day before surgery; T1, on the 4th postoperative day; T2, on the 9th postoperative day; T3, at the 10th week postoperatively.

## Data Availability

Data is available on request from the corresponding author.
